# The Efficacy and Safety of Antiplatelet Therapy in Patients With Acute Coronary Syndrome: A Scoping Review

**DOI:** 10.7759/cureus.49631

**Published:** 2023-11-29

**Authors:** Tanusha Winson, Prithvi Basu Roy, Vitrag N Tejani, Sukhmeet S Dhillon, Nanush Damarlapally, Nia Uswanti Binti Usman, Binay K Panjiyar

**Affiliations:** 1 Internal Medicine, AIMST University, Sungai Petani, MYS; 2 Cardiology, KPC Medical College and Hospital, Kolkata, IND; 3 Pharmacology, Dr. N. D. Desai Faculty of Medical Science and Research, Dharmsinh Desai University, Kheda, IND; 4 Internal Medicine, Parul Institute of Medical Sciences and Research, Parul Sevashram Hospital, Parul University, Vadodara, IND; 5 Internal Medicine, Baba Farid University of Health Sciences, Patiala, IND; 6 Health Sciences, Coleman College of Health Sciences, Houston, USA; 7 Internal Medicine, Universitas Brawijaya, Kajang, MYS; 8 Cardiology, Harvard Medical School, Boston, USA; 9 Internal Medicine, California Institute of Behavioral Neurosciences & Psychology, Fairfield, USA

**Keywords:** efficacy and safety, heart attack, acs ( acute coronary syndrome ), dual-antiplatelet therapy (dapt), antiplatelet drugs, antiplatelet therapy

## Abstract

Cardiovascular disease, predominantly acute coronary syndrome (ACS), is the leading cause of death for both men and women. For decades, this has been a global healthcare challenge. The main reason for thrombus formation in the coronary arteries is platelet accumulation as part of an inflammatory reaction. The efforts to combat this process of platelet aggregation have led researchers to discover antiplatelet drugs, which have been a keystone in treating cardiovascular diseases related to arterial thrombus formation. Antiplatelet drugs inhibit various platelet responses and help mitigate atherothrombosis, thereby playing a major role in both primary and secondary prevention of ACS.

This study employs a scoping review approach to recapitulate the data in the existing literature regarding the efficacy and safety of antiplatelet therapy in patients with ACS. By searching a total of 14,882 journals that were published between 2013 and July 26, 2023, 10 papers were selected for in-depth analysis. We conducted this literature search by using PubMed and Google Scholar databases and adhered to the Preferred Reporting Items for Systematic Reviews and Meta-Analyses (PRISMA) and the corresponding PRISMA Extension for Scoping Reviews in performing this review.

The review findings revealed that the current approach of using antiplatelet agents in ACS is safe and efficient, provided that bleeding risk assessment is conducted and any prior contraindications are recognized before administering the drugs.

Ethical approval was not required for this review as it involved secondary data collection from published journals. The findings of this scoping review will be published in peer-reviewed journals and presented at conferences.

## Introduction and background

Ischemic heart disease (IHD) and acute coronary syndrome (ACS) are among the primary causes of death at a younger and economically productive age in low- and middle-income countries (LMICs) than in high-income nations [1]. ACS is the primary cause of death in the Asia-Pacific region, accounting for more than 50% of the global burden [2]. The root cause of coronary artery disease (CAD) has been proven to be atherosclerosis, a chronic inflammatory process [3]. Arterial thrombosis following atherosclerotic plaque rupture or erosion is the primary trigger of ACS [4,5].

ACS occurs when blood flow to the heart muscle gets interrupted. ACS can occur if a blockage forms in the coronary artery, either partially or fully. Based on electrocardiogram (ECG) results, acute myocardial infarction (AMI) is classified into two types: ST-segment elevated myocardial infarction (STEMI) and non-ST-segment elevated myocardial infarction (NSTEMI). A complete coronary artery occlusion causes STEMI, whereas NSTEMI patients have a partial blockage and no apparent ST-elevation on their ECG. STEMI patients are at a higher risk of death than NSTEMI patients [6]. Platelet adhesion, activation, and aggregation play an essential role in the initial phase of thrombosis soon after an atherosclerotic plaque ruptures, breaks, or erodes. This process constitutes a significant pathogenic phase in the development of ACS-unstable angina (UA), STEMI, and NSTEMI. Antiplatelet therapies play an integral role in treatment in the setting of ACS since atherothrombotic events are fundamentally platelet-driven processes [7,8]. Therefore, it is essential to conduct studies to assess the efficacy and safety of antiplatelet therapy in ACS.

There are currently three groups of antiplatelet medications approved for treating and preventing ischemic events in ACS: cyclooxygenase (COX)-1 inhibitors such as aspirin; adenosine diphosphate (ADP) P2Y12 receptor antagonists like clopidogrel, prasugrel, ticagrelor, and cangrelor; and glycoprotein IIb/IIIa inhibitors (GPI) such as abciximab, eptifibatide, and tirofiban [8-11]. Aspirin, a COX inhibitor, was first used in cardiovascular prophylaxis in the 1980s, and the subsequent introduction of ADP receptor P2Y12 inhibitors not only provided an alternative for aspirin-intolerant patients but also the potential for high-intensity platelet inhibition due to concurrent blockade of both the COX and ADP-dependent pathways [12]. Aspirin acetylates an essential serine residue in the active region of the COX-1 isoenzyme, irreversibly inhibiting the COX activity of platelets and reducing thromboxane-dependent activation of platelets [12]. The platelet P2Y12 receptor binds ADP, activating the platelet glycoprotein IIb/IIIa receptor through Gi protein, increasing platelet degranulation, thromboxane generation, and aggregation of platelets [12]. Because of its importance in platelet activation, the P2Y12 receptor is a prominent target for antiplatelet drugs. Ticlopidine, a thienopyridine of the first generation, was developed in 1972 and approved as an antiplatelet agent in 1978. Despite its beneficial antiplatelet action, ticlopidine causes uncommon but possibly catastrophic adverse effects such as aplastic anemia, neutropenia, and thrombotic thrombocytopenic purpura, which call for constant hematological monitoring [13].

Clopidogrel, a second-generation thienopyridine with no hematological side effects of ticlopidine, was licensed for usage in 1997. Clopidogrel swiftly replaced ticlopidine due to its similar efficacy and better safety profile. Subsequently, other P2Y12 receptor blockers such as prasugrel and ticagrelor were introduced, which fix clopidogrel's disadvantages such as slow onset of action and variation among individuals in antiplatelet response [14]. Thienopyridine derivatives block the P2Y12 receptor irreversibly, resulting in antiplatelet action. Conversely, ticagrelor is a novel chemical family with a faster onset of effect with consistent inhibition of platelets that requires no hepatic metabolism and leads to reversible blockage of the P2Y12 receptor [15].

The last common pathway leading to platelet aggregation is mediated by fibrinogen binding to the glycoprotein IIb/IIIa receptor on the surface of platelets [16]. Glycoprotein IIb/IIIa receptor antagonists attach to the glycoprotein IIb/IIIa receptor, preventing fibrin from binding to the receptor [17]. Glycoprotein IIb/IIIa receptor inhibitors that have received clinical approval include abciximab, eptifibatide, and tirofiban. Their usage is typically not suggested for low- to moderate-risk individuals or patients with a conservative approach. In contrast, they provide the most significant benefit to those with severe ACS and undergoing percutaneous intervention (PCI) [18].

This review evaluates the available evidence regarding the various antiplatelets currently being used to treat ACS by addressing the following question: do the currently available antiplatelet agents benefit patients with ACS?

Rationale

Over the years, the use of antiplatelets in ACS has become increasingly prevalent, and hence being aware of the mode of action, safety, interactions with other medications, and adverse reactions of these widely used drugs will help guide future interventions, and this knowledge will serve as an excellent source of information for other medical professionals and researchers in addition to illuminating the gaps and current discrepancies in the understanding regarding the safety and efficacy of these drugs. 

Objectives

This scoping review's foremost objective is to analyze various review articles to precisely (1) identify how the various antiplatelets work, including their uses, mechanism of action, drug interaction, and adverse effects; and (2) identify the efficacy and safety of antiplatelets in ACS, and determine if the antiplatelet agents currently in use benefit patients with ACS.

## Review

Methods

**Figure 1:**
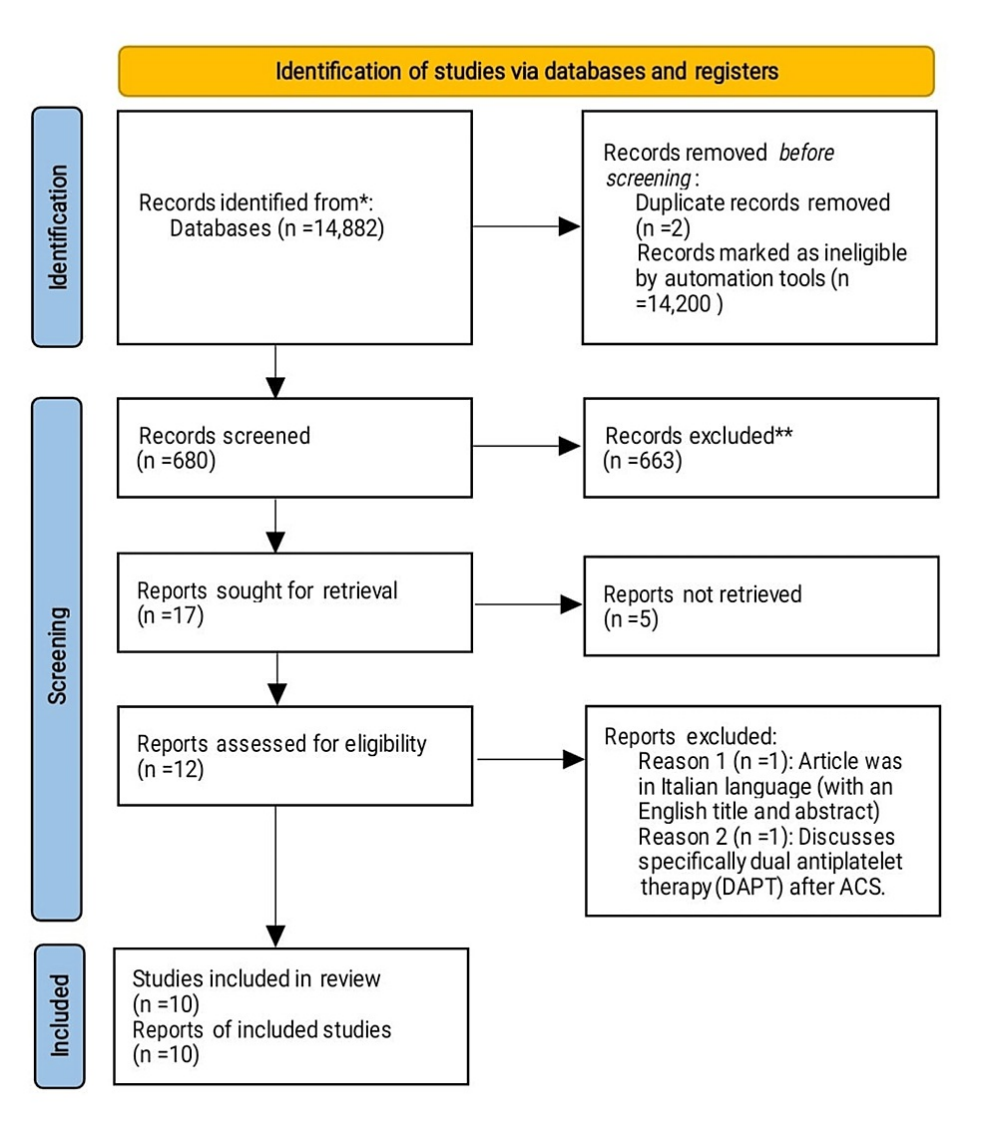
PRISMA flow diagram illustrating the search strategy and study selection process PRISMA: Preferred Reporting Items for Systematic Reviews and Meta-Analyses; ACS: acute coronary syndrome

This scoping review focuses on clinical studies on the use of antiplatelet agents in ACS. We excluded animal studies and publications that only discussed the methodology related to antiplatelet agents without presenting clinical data. We adhered to Preferred Reporting Items for Systematic Reviews and Meta-Analyses (PRISMA) guidelines [19] 2020 (Figure 1) in conducting this review. We only used data collected from published papers, and hence ethical approval was not required.

Systematic Literature Search and Study Selection

A thorough search was conducted on the databases PubMed, Medline, and Google Scholar to look for pertinent papers. We looked for research cited in reviews, editorials, and commentaries on PubMed. We also diligently searched for further papers that met our inclusion criterion. We independently assessed a list of abstracts for inclusion based on specific criteria. The criteria included the efficacy and safety of antiplatelet drugs in ACS and a clearly described clinical cohort in the study. We excluded review papers and animal studies. 

Inclusion and Exclusion Criteria

We established specific eligibility criteria for including and excluding papers to achieve our study goals. Our criteria are summarized in Table 1. 

**Table 1:** The criteria adopted for the inclusion of studies

Inclusion criteria	Exclusion criteria
(a) Human studies	(a) Animal studies
(b) Published from 2013 to 2023	(b) Methodological studies that only explain programming details
(c) English text	(c) Non-English texts
(d) Gender: all	(d) Age ≥45 years
(e) Age: 45+ years	(e) Studies involving clinical data other than cardiovascular disease
(f) Free papers	(f) Papers that needed to be purchased

Search Strategy

A thorough literature review was conducted based on the population, intervention/ condition, control/comparison, and outcome (PICO) criteria. The search involved relevant keywords such as "antiplatelet therapy", "efficacy", "safety", and "acute coronary syndrome" on databases like PubMed (including Medline), and Google Scholar. The medical subject heading (MESH) approach for PubMed (including Medline) and Google Scholar, as detailed in Table 2, was employed to develop a comprehensive search strategy.

**Table 2:** The search strategy, search engines used, and the number of results elicited

Database	Search strategy	Results
PubMed	(((((Antiplatelet therapy[Title/Abstract]) OR (Efficacy of antiplatelet therapy[MeSH Terms])) OR (Safety of antiplatelet therapy[MeSH Terms])) OR (Antiplatelet on Acute Coronary Syndrome[MeSH Terms])) OR (Antiplatelet on Heart Attack[MeSH Terms])) AND (("2013/01/01"[Date - Publication] : "3000"[Date - Publication]))	12,020
PubMed (with filters)	(((((Antiplatelet therapy[Title/Abstract]) OR (Efficacy of antiplatelet therapy[MeSH Terms])) OR (Safety of antiplatelet therapy[MeSH Terms])) OR (Antiplatelet on Acute Coronary Syndrome[MeSH Terms])) OR (Antiplatelet on Heart Attack[MeSH Terms])) AND (("2013/01/01"[Date - Publication]: "3000"[Date - Publication])) AND ((y_10[Filter]) AND (ffrft[Filter]) AND (clinicaltrial[Filter] OR meta-analysis[Filter] OR randomizedcontrolledtrial[Filter] OR review[Filter] OR systematicreview[Filter])) Filters applied: Free full text, Clinical Trial, Meta-Analysis, Randomized Controlled Trial, Review, Systematic Review, in the last 10 years, Humans, English, Female, Male, Middle Aged + Aged: 45+ years, MEDLINE	582
Google Scholar	(((Antiplatelet* OR Efficacy OR Safety) OR Acute Coronary Syndrome OR Heart Attack) AND Antiplatelet Therapy) Filters applied: Studies from 2013 to 2023, Review articles	14,300

Quality Appraisal

We utilized various quality assessment tools to ensure the validity of the articles we shortlisted. We employed the PRISMA checklist and Cochrane risk-of-bias tool for randomized trials. Non-randomized clinical trials were evaluated using the Newcastle-Ottawa scale. We assessed the quality of qualitative studies, as shown in Table 3, by using the critical appraisal skills program (CASP) checklist.

**Table 3:** Quality appraisal tools used PRISMA: Preferred Reporting Items for Systematic Reviews and Meta-Analyses

Tools used	Type of studies
Cochrane risk-of-bias tool	Randomized controlled trials (RCT)
Newcastle-Ottawa scale	Non-RCTs and observational studies
PRISMA checklist	Systematic reviews

Results

We retrieved 14,882 articles from the three chosen databases PubMed, Medline, and Google Scholar. Following an in-depth analysis of each publication and the application of specific criteria, 14,202 papers were ultimately excluded, including two duplicate papers. We screened the remaining 680 papers and excluded 663 papers due to unsatisfactory titles and abstracts. From the remaining 17 papers, we filtered out another five papers because their content did not meet our inclusion standards. Of the 12 papers left, we excluded one written in the Italian language and another one that specifically discussed dual antiplatelet therapy (DAPT). The final step involved a rigorous quality review of the remaining 10 papers, all of which satisfied our standards. Our final review includes these 10 papers, which are summarized in Table 4.

**Table 4:** Summary of the findings of the selected papers ACS: acute coronary syndrome; PCI: percutaneous coronary intervention; DAPT: dual antiplatelet therapy; CAD: coronary artery disease

Author/year	Country	Study design	Database used	Conclusion
Dash/2015 [20]	India	Review	PubMed	Antiplatelet therapy, like aspirin and clopidogrel, has a well-established therapeutic function in treating ACS, although recent research shows that prasugrel and ticagrelor have distinct advantages over clopidogrel. Numerous new antiplatelet drugs still need to be clinically validated to help establish the most efficient treatment regimen for patients with ACS or undergoing PCI
Franchi and Angiolillo/2014 [21]	USA	Review	PubMed and www.clinicaltrials.gov website	In several clinical trials, antiplatelet medication is crucial in managing and secondary prevention of ischemic episodes in individuals with ACS
Passacquale et al./2022 [12]	UK	Invited review	PubMed	Antiplatelet treatment is a vital component of cardiovascular prevention, provided the treatment is personalized for each patient based on a detailed evaluation of their thrombotic vs. bleeding risk
Kolandaivelu and Bhatt/2011 [22]	USA	Review	PubMed, Google Scholar, and Scopus	With proper medication adherence, antiplatelet therapy plays an essential role in preventing coronary heart disease
Lee/2015 [15]	Korea	Review	PubMed	Compared to aspirin and clopidogrel, aspirin and novel P2Y12 receptor blockers have a lower risk of cardiovascular complications following ACS. However, long-term DAPT raises the risk of severe bleeding, necessitating a careful balancing act between the risk of bleeding and anti-ischemic benefit
Packard et al./2012 [23]	USA	Review	Google Scholar, PubMed	In those suffering from CAD and ACS, dual antiplatelet treatment with aspirin plus clopidogrel or prasugrel has been proven efficient
Sharma et al./2020 [24]	India	Review	Google Scholar and PubMed	According to the most recent guidelines, patients with ACS are encouraged to use DAPT for 12 months. Nonetheless, as in any medical situation, an individual evaluation is necessary
Tendera and Wojakowski/2003 [25]	Poland	Review	PubMed, Google Scholar, and Scopus	Double antiplatelet therapy is more successful than aspirin alone in preventing stent thrombosis in coronary stenting
Udell et al./2015 [26]	Canada	Systematic review and meta-analysis	OVID Medline and Cochrane	DAPT treatment exceeding one year delivers a significant decrease in critical cardiovascular complications. It has to be considered in patients with past myocardial infarction who are at minimal bleeding risk
Tisdale/1998 [17]	USA	Journal article	PubMed	Patients with an acute previous myocardial infarction, history of stroke or prior transient ischemic attack, unstable angina, and various vascular illnesses experienced considerably fewer vascular-related complications due to the use of antiplatelet medication

Discussion

Appropriate treatment with high efficacy and safety is necessary to enhance survival and outcome in patients with ACS. Based on the currently approved antiplatelet drugs, aspirin has been used as the core medication in patients with ACS. It acts by irreversibly blocking COX1, reducing the synthesis of thromboxane A2 (TXA2), which binds to thromboxane and prostaglandin endoperoxide (TP) receptors, and subsequently resulting in changes to platelet shape and increases platelet recruitment and aggregation [27]. As a result, aspirin reduces TP receptor-mediated platelet activation and aggregation [27]. When aspirin was first discovered, its use in ischemic heart diseases was debatable, apart from concerns regarding its side effects, such as peptic ulcer and gastrointestinal bleeding, especially when administered in large doses. Administering aspirin 1,000 mg/day could not prevent death in patients with prior myocardial infarction, according to the first major randomized study on the topic: the Aspirin Myocardial Infarction Study (AMIS) [28]. Moreover, adverse drug reactions were more prevalent in the aspirin group compared to the placebo group. However, a major, randomized study called the International Study of Infarct Survival (ISIS)-2 found that 160 mg/day of aspirin significantly reduced vascular mortality in patients with acute myocardial infarction by 23% compared to placebo [29]. The most widely used antiplatelet drug now is aspirin. For individuals with existing atherosclerotic coronary artery disease, current guidelines recommend using low-dose aspirin (75-325 mg/day) continuously [30].

The next group of antiplatelet drugs is called thienopyridines, P2Y12 inhibitors. An essential platelet agonist, ADP works by activating P2Y1 and P2Y12 receptors on the platelet plasma membrane. Of these two, the P2Y12 route exerts the most impact on platelet aggregation and stabilization [31]. Hence, blocking the action of the P2Y12 receptor exerts a great antiplatelet function. Ticlopidine, a first-generation drug, was discovered but had to be replaced with clopidogrel (second-generation) and the newer drugs prasugrel and ticagrelor due to the severe side effects [13] of ticlopidine. Cytochrome p450 isoforms first mediate the process of double oxidation of the prodrug clopidogrel in the liver before it becomes metabolically active. It selectively and irreversibly blocks the ADP P2Y12 receptor, ensuring that the effects remain for 7-10 days of platelet lifetime [32,33]. Given the fact that one group of antiplatelet agents can be so efficacious in treating and preventing cardiac ischemic events, how about combining two groups of drugs?

Dual antiplatelet therapy (DAPT) combines aspirin and a P2Y12 antagonist, namely clopidogrel. In individuals with ACS, DAPT has been proven to reduce recurrent major ischemic episodes [34]. The usual one-year prescription for DAPT is based on findings from significant early antiplatelet studies like CURE that analyzed the course of P2Y12 inhibition. Premature DAPT withdrawal has been associated with incidents of late stent thrombosis in patients with first-generation drug-eluting stents (DES). Hence, it was empirically pointed that DAPT be continued for up to 12 months in these individuals [35]. The CURE study identified that among 12,262 patients with NSTE-ACS, there was a significant decrease in the primary composite endpoint [cardiovascular (CV) death, non-fatal MI, or stroke] in the clopidogrel combined with aspirin arm versus aspirin alone at 12 months (9.3 vs. 11.4%, RR: 0.80, 95% CI: 0.72-0.90, p=0.001), with an increased risk of major bleeding (3.7 vs. 2.7%, RR: 1.38, p=0.001). Nevertheless, there was no substantial difference in mortality rates between treatment groups (5.1 vs. 5.5%, HR: 0.93, 95% CI: 0.79-1.08) [36]. Meanwhile, a meta-analysis by Udell et al. [26] stated that compared with aspirin alone, prolonged DAPT beyond one year produced a 22% relative and 1.1% absolute risk reduction for severe cardiovascular side effects throughout a mean 31-month follow-up involving more than 33,000 high-risk patients with stabilized MI. Therefore, it is recommended to use clopidogrel in combination with aspirin to treat ACS since it reduces long-term adverse events in patients with and without PCI [37].

Prasugrel, a third-generation thienopyridine, is an oral prodrug. In contrast to clopidogrel, prasugrel only needs a single oxidative step dependent on CYP to produce the active metabolite. Thus, prasugrel inhibits platelets more quickly, effectively, and consistently than clopidogrel because it is metabolized by esterases rather than CYP enzymes [38]. A phase 3 trial involving 13,608 patients [TRITON-TIMI 38 (Trial to Assess Improvement in Therapeutic Outcomes by Optimizing Platelet Inhibition with Prasugrel-Thrombolysis in Myocardial Infarction 38)] showed that prasugrel (60 mg loading dose and a 10-mg daily maintenance dose) significantly decreased death, myocardial infarction, and stroke in patients with ACS who were scheduled for PCI compared to the recommended clopidogrel doses (300 mg loading dose and 75 mg per day), but with a higher risk of severe bleeding, including life-threatening hemorrhage [39-41]. However, prasugrel's net advantage compared to clopidogrel was particularly evident in individuals with STEMI [42].

The cyclopentyl-triazolopyrimidine class drug ticagrelor is a reversible P2Y12 antagonist that, in contrast to thienopyridines, is active on its own and is not dependent on hepatic metabolism to exert its pharmacological action. On the other hand, the drug undergoes extensive cytochrome-mediated oxidation, which results in the generation of an active by-product that also accounts for the beneficial effects [43]. Ticagrelor has a more rapid onset, as well as robust, and reliable antiplatelet activity than clopidogrel and a quicker offset of action [44].

Glycoprotein IIb/IIIa (GP IIb/IIIa) receptor inhibitors such as abciximab, eptifibatide, and tirofiban provide the most significant benefit only in those with severe ACS undergoing PCI and not for those who are at low- to moderate-risk [18], mainly not only due to its lack of beneficial effect but higher risk of mortality. There is a need for dose titration because high levels of platelet inhibition from oral GP IIb/IIIa receptor antagonists were linked to a very high incidence of major (11.8%) and minor (70%) bleeding events. This effect may result in long-lasting insufficient platelet inhibition, antagonist-induced prothrombotic activity, and a deficiency of protective properties [45,46]. However, most of the insufficient therapeutic advantages of oral GP IIb/IIIa receptor antagonists occurred with first-generation drugs like sibofiban, orbofiban, and xemilofiban may have been due to their distinct pharmacokinetics when they were previously administered for individuals with established IHD. Therefore, newly developed oral medications in this class, like roxifiban, were created and put through "response to dose" and "effectiveness" studies. Roxifiban is an oral non-peptide platelet GP IIb/IIIa receptor blocker that has excellent stability of plasma concentrations throughout dosage, a more potent blocking effect on tissue factor-induced platelet aggregation, higher affinity, longer elimination half-life (four to six days), and a more gradual breakdown from resting and activated platelets. These factors, taken together, could mitigate the side effects caused by the drug [47].

To sum up, all these classes of antiplatelet drugs evidently benefit those with IHD, especially ACS. While these agents are used to treat the condition, taking them to prevent the disease is also likely possible. As demonstrated by the PolyIran study, aspirin-containing polypill approaches have successfully hindered major cardiovascular events in low- and middle-income countries [48]. In contrast, Antithrombotic Trialists' collaboration in 2009 raised concerns about aspirin's net benefit in primary prevention due to an observed elevated chance of severe extracranial and gastrointestinal bleeding adverse effects despite having just a mild protection towards vascular events, as recorded in an extensive meta-analysis carried out by them [49]. Therefore, medical professionals must carefully assess the risk of bleeding in each patient and tailor the treatment accordingly to prevent unwanted complications.

Limitations

This systematic review has a few limitations. We included only human studies, specifically focused on those at least 45 years of age, and limited our research to English publications published in the past 10 years. Additionally, we only analyzed free articles and focused our research on English language papers regarding the efficacy and safety of antiplatelet therapy in ACS. Further studies are required to achieve more precise results and gain deeper insights into the topic.

## Conclusions

In the rapidly evolving field of antiplatelet therapy, it could be a challenging prospect to thoroughly analyze and make sense of all the studies and research on the efficacy and safety of each antiplatelet medication, either as monotherapy or combination therapy in patients with ACS. As a growing number of novel medications are introduced, there could be a paradigm shift in how antiplatelet medications are administered to patients with ACS. Nevertheless, studies have successfully proven the favorable effects of antiplatelet treatment on ACS patients, with remarkable safety and efficacy, provided that the prescribed medication and dose have been customized individually, following a meticulous evaluation of the patient's risk of bleeding.
